# Laying the groundwork and designing for trust: why research protocols matter

**DOI:** 10.1186/s41077-026-00430-1

**Published:** 2026-03-10

**Authors:** Ellen Davies

**Affiliations:** Adelaide Health Simulation, College of Health, Adelaide University, Adelaide, Australia

## Abstract

**Background:**

The processes we use to conduct research have evolved over decades and centuries. These evolutions have been shaped by scientific advancements in practice, theory and technology, by community expectations, and through reflections on what we consider to be good research practice. One fundamental process that is not well represented in the public domain is the research protocol, particularly in health simulation and health professions education research.

**Discussion:**

Research protocols provide detailed information about why and how research is being conducted. At present, it is not common practice for these documents to be made available to the broader community before data collection commences. Nor is it common practice for protocols to be reviewed alongside final project reports at the peer-review stage. This paper describes and discusses many of the reasons that a priori research protocols are increasingly being made publicly available through peer-reviewed publication or registration on open science platforms. These include the reduction of research waste, the increase in trust we can engender through transparency, and the opportunities we have to learn through the processes of conducting research, not just from the findings.

**Conclusion:**

Over time, and as a society, there have been important opportunities to consider the standard of research to which we aspire. We have an opportunity here to consider the foundations from which we are working, and to elevate the quality of our projects through strengthening these foundations. The careful design and publication and/or registration of a priori protocols is perhaps the next step we can take to increase the quality of our work and our standing in the scientific and health communities.

## Introduction

Research protocols have evolved alongside advances in science, ethics and regulatory frameworks. Protocols outline projects’ aims and research questions, methods for data collection, approaches for data analysis and project reporting, and importantly can be made publicly available *prior* to any data collection [1, 2]. Ideally, protocols also provide justification for projects’ use of proximal resources (material goods, participant and researcher time) and distal resources (peer-review and publication time and costs).

Registered and published protocols play a pivotal role in the scientific process: they guard against poor quality scientific enquiry, reduce research waste, enhance public and scientific community trust and reduce publication bias [3, 4]. And yet, despite these recognised benefits, their adoption in health simulation research, and health professions education research more broadly, is scarce.

The publication of literature review protocols (for example, for systematic and scoping reviews) has become more prevalent in our field, and in many others [1]. Indeed, we are seeing growing audience and peer-reviewer expectations to access registered and/or published a priori protocols for this type of work. But this expectation has not been widely transferred to primary research (i.e. projects that are original research projects where new data is sourced and analysed to address one or more research questions).

In this discussion paper, I’d like to convince you that research protocols matter, that they should be registered and/or published prior to data collection for all evidence synthesis and primary research projects and that the research processes and outputs generated from our field will be much more valuable when we do so. This discussion paper provides an overview of some of the fundamental reasons *publicly* and *prospectively* available protocols add value and credibility to research projects, outlines commonly used protocol guidelines, considers the relationship between research protocols and reporting guidelines and offers some reflections on what it may mean for health simulation and health professions education research when it becomes accepted and expected practice to make research protocols publicly available.

## What is a protocol and why should we care?

A protocol is a document that lays the groundwork for primary research projects and evidence syntheses (i.e. literature reviews). It declares in detail what we are interested in; how others have pursued the same or similar interests; how we anticipate our planned project will add to, or refute, current scientific understandings; how we will recruit participants (where relevant); how we will collect and analyse data and; how we intend to report our findings [3, 5, 6].

For those who have applied for permission to undertake a research project from a human ethics review committee or board, you will be familiar with many structural elements of a protocol. However, the protocol submitted to the ethics committee is not publicly available when the project receives approval to proceed, it is not necessarily critiqued by content experts who can comment on the worth, rigour, feasibility and appropriateness of the study, nor is it available as a resource for future research on the same or similar topic.

The impetus to make research protocols publicly accessible can be well understood in the context of the historical evolution of research ethics and trial regulation, which have largely emerged in response to past abuses of participants and increasing scientific complexity [7, 8]. Ethical frameworks to explicitly and purposefully protect research participants were constructed after revelations of unethical human experimentation, such as those documented in the Nuremberg trials post-World War II [8]. Landmark documents, like the Declaration of Helsinki (first published in 1964, and revised since this time) established ethical principles requiring research transparency and informed consent [8]. The late twentieth century saw increased regulatory attention for research conduct, including the establishment of ethics review committees (also known as Institutional Review Boards) and informed consent expectations. Concurrently, the demand for greater transparency and accountably in research gained momentum with international guidelines emphasising the importance of pre-specifying research methods to prevent selective reporting and bias [9]. Publication of research protocols is now recognised as a primary prevention mechanism to support researchers to fulfil ethical principles and to counter academic misconduct – be that intentional or unintentional.

### Rigorous scientific planning for rigorous scientific work

The design and development of a protocol is fundamental to the process of conducting rigorous research that contributes valid knowledge to a field. *Rigour* refers to the systematic, transparent, and thorough approach used in both the design and execution of a study to minimise bias, enhance credibility and ensure trustworthy findings [10–12]. Rigorously conducted research projects of all types require careful planning, clear justification of methodological choices, consistent and explicit procedures, detailed reporting and reflexivity, whereby researchers recognise and report their influence on research processes and outcomes [13]. In the absence of rigorous project planning, ad hoc decisions can be made that significantly alter who is involved in the project (participants), how they are involved, what data is collected, how those data are analysed and how they are interpreted. This drift can be small or seismic, resulting in projects that are no longer well-aligned with initial questions and aims, and findings that may not withstand scrutiny. A publicly available protocol guards against scientific enquiry that is not conducted with the rigour that is required to produce defensible and useful findings.

### Transparency and accountability

There exists a valid and reasonable community expectation that researchers are transparent and accountable for the work they undertake, regardless of whether this is qualitative, quantitative, mixed-method or evidence synthesis work. Transparency allows project methods, outcomes and interpretations to be critiqued, reduces risk of scientific misconduct and improves scientific and community trust in researchers and their outputs [9, 14, 15]. Pre-registering or publishing protocols is a powerful mechanism for researchers to demonstrate transparency in their research endeavours, and forces accountability at each step of the research process. It minimizes the risks of selective outcome reporting (for example where only data that supports a certain hypothesis or position are published) and post hoc changes to research questions, aims, methods and approaches to data analysis [1]. Protocol-based research is less prone to fraud and error, improving the confidence of both the scientific community and the public [1].

### Reducing risk of research waste

*Research waste* is a term that refers to ‘research outcomes with no societal benefits’ [16], p. 495). Several commentators have used the term to describe the human, physical and institutional resource wastage that occurs when there are avoidable failures at different stages of the research process that lead to irrelevant, indefensible, misleading and/or incorrect findings [17–19]. Economically, billions of dollars are lost annually to poorly conducted scientific research [18].

Decisions (or lack thereof) that are made at each stage of a project may result in research waste. Poor understanding and integration of past relevant research, poor design of the research question, inappropriate and poorly aligned methods for addressing research questions, inaccessible data, inappropriate decisions relating to data analysis and poor project reporting are all examples of problems that ultimately lead to research waste [15, 16, 19, 20]. Most of these problems could be addressed (or at the very least minimised) with good planning and the development of a robust protocol.

### Learning from the process, not just the outcomes

The *processes* of developing and adhering to research and review protocols provide some of the richest opportunities for learning about our topic, our field of interest and our research skillset. While it is exciting to *know the answer,* or to *come to a conclusion*, the work we do in research is much more than producing findings.

The breadth of research conducted in health simulation and health professions education is impressive. Quantitative, qualitative, mixed- and multi methods and evidence synthesis approaches to enquiring into simulation and education practice, outcomes, process and experiences have all be used [21]. Acknowledging the benefits of this heterogeneity and the evidence base that has been established through a myriad of research methodologies and methods, there is scope for refinement of the questions we are asking as a field, and there is scope for improvement in the quality of projects that are being conducted [22–24].

When we allow other’s from beyond our research team to review our protocols, we have an opportunity to learn from their experiences and expertise. Whether this is a formal process (i.e. peer review for publication), or an informal process (for example a step-back consultation process [25]), the invitation for critique and feedback can strengthen a project’s relevance to the field and the rigour with which it is undertaken.

When other teams’ protocols are available to us, we can learn about how other people are approaching the same or similar topics or methods of interest. Through seeing the details that are not always reported in final project reports, we can learn how different methodologies and methods are being crafted to address research questions that align with our own interests, providing opportunity to sharpen and refine our approaches and potentially to connect with other teams for support and to collaborate on future projects.

With reference to the poor conduct of research, and institutional drivers for publication *quantity over quality*, Altman wrote in his 1994 essay: ‘we need less research, better research, and research done for the right reasons [17], p. 283). Thirty years later, we still need to hear and engage with this message.

## Protocol development guidance

Guidelines to support the development of research protocols exist for different types of research. For example, the Standard Protocol Items: Recommendations for Interventional Trials (SPIRIT) guidelines were designed specifically for clinical trials [26, 27], and the Preferred Reporting Items for Systematic Reviews and Meta-Analyses (PRISMA) group published guidelines specific to developing systematic review protocols [28]. There are numerous types of research that do not have specific protocol development guidelines. Qualitative research and mixed methods research are largely un-chartered territories with respect to protocol guidelines. In the absence of dedicated protocol development guidelines for different types of research (for example, qualitative and mixed-method research, we have available to us reporting guidelines that provide information about the details required to adequately report a research project. Some of these provide information about what needs to be in a protocol. Table 1 provides examples of relevant protocols, corresponding or complementary reporting guidelines, extracted information that relates to the content required in a well-constructed protocol and examples of publications that have used these guidelines in health simulation and/or health professions education research.

**Table 1:** Examples of relevant protocol and reporting guidelines

Type of research activity	Protocol guideline/checklist examples	Reporting guideline/checklist examples	Features of protocol guideline/checklist OR guidance from reporting guideline	Examples of *registered* health simulation &/or health professions education protocols^†^	Examples of *published* health simulation &/or health professions education protocols^†^
Primary research					
Quantitative – Randomised Controlled Trials (RCTs)	SPIRIT 2025 [26]	CONSORT reporting guidelines STROBE reporting guidelines	SPIRIT guidelines have a detailed checklist items organised under headings: administrative information; open science; introduction; methods: patient and public involvement, trial design; methods: participants, interventions and outcomes; methods: assignment of interventions; methods: data collection, management, and analysis; methods: monitoring; ethics	“A Pilot Evaluation of an Online Mindfulness-Based Compassion and Self-Caring Intervention Program (MSELF) on University Students in Helping Professionals Discipline: A Cross-Geographical Randomized Controlled Trial” [29]	“Self-guided versus facilitator-guided debriefing in immersive virtual reality simulation: Protocol for a randomized controlled non-inferiority trial assessing teamwork skills in medical students” [30]
Quantitative – other	SPIRIT 2025 [26]	STROBE extension guidelines for simulation [31]		“Identifying opportunities in improvement of learning outcome in simulation-based training of health care personnel—A Danish Setting” [32]	“A study protocol for interprofessional collaborative, digital, and sustainability training in primary healthcare: the REALISE study” [33]
Qualitative	Refer to BQQRQ, SRQR and other qualitative research reporting guidelines	BQQRQ [34] SRQR [35]	BQQRQ provides advice on practices to consider, practices to avoid and notes for reviewers of qualitative research studies SRQR provides reporting guideline items in a checklist organised under headings: title and abstract; introduction; methods; results/findings; discussion; other	“Principles and frameworks for testing cognitive aids in simulation: a qualitative study protocol” [36]	“Functions and roles of embedded participants in health simulation – an exploratory qualitative observational study protocol” [37]
Mixed/multi methods	Refer to reporting guidelines, for example MMR-RHS	MMR-RHS [38]	MMR-RHS is a 20-item reporting guideline specifically designed for projects where distinct quantitative and qualitative methods are used together and emphasises integration of these methods throughout	“Evaluating the scope of virtual reality (VR) in podiatric surgery education: Mixed method” [39]	“Evaluation of the feasibility and impacts of in situ simulation in emergency medicine-a mixed-method study protocol” [40]
Quality improvement and simulation-based quality improvement projects	Refer to SQUIRE 2.0 and SQUIRE-SIM reporting guidelines	SQUIRE 2.0 [41] SQUIRE-SIM [42]	SQUIRE 2.O provides reporting guideline items in a checklist organised under headings: title and abstract; introduction; methods; results; discussion; other information	“Emergency Ultrasound Minimal Imaging Criteria Quality Improvement” [43]	“Implementing resilience engineering for healthcare quality improvement using the CARE model: a feasibility study protocol” [44]
Literature reviews					
Systematic literature review	PRISMA-P guidelines [28]	PRISMA 2020 statement [45]	PRISMA-P and PRISMA are well aligned by design. These checklist items are organised under headings: administrative information; introduction; methods; results; discussion; other information. The PRISMA statement also includes a checklist for abstracts	“Virtual reality simulation in obstetric training for nursing students” [46]	“The Impact of Simulation Facilitation on Learning Outcomes – A Systematic Review Protocol” [47]
Scoping review	PRISMA-P guidelines [28] JBI best practice guidelines for scoping review protocol development [48]	PRISMA-ScR [49]	PRISMA-P and PRISMA extension for scoping reviews are well aligned by design. These checklist items are organised under headings: administrative information; introduction; methods; results; discussion; other information. The PRISMA statement also includes a checklist for abstracts	“Harnessing lived experience in health professions simulation-based education: a scoping review protocol” [50]	“Simulated clinical placements in health care education: a scoping review and evidence and gap map protocol” [51]
Other forms of evidence synthesis (for example, mixed methods systematic review, qualitative systematic review, umbrella reviews)	Refer to JBI Manual for Evidence Synthesis [52] PRISMA-P guidelines [28]	Examples include: PRIOR [53] PRISMA statement [45]	Detailed statements for what should be included in the final report provided in reporting guidelines. Relevant extracts from PRIOR [53]: Item 23a: Provide registration information for the overview of reviews, including register name and registration number, or state that the overview of reviews was not registered Item 23b: Indicate where the overview of reviews protocol can be accessed, or state that a protocol was not prepared Item 23c: Describe and explain any amendments to information provided at registration or in the protocol. Indicate the stage of the overview of reviews at which amendments were made	“Supporting the wellbeing of health and care students during placements: a mixed-methods systematic review protocol” [54]	“Simulation-based education to support new graduate nurses during transition to practice in critical care: a mixed methods systematic review protocol” [55]
Consensus development					
Consensus development projects (e.g. Delphi studies, nominal group technique, taxonomy development)	Refer to ACCORD reporting guideline	ACCORD reporting guideline [56]	Relevant extracts from reporting guidelines: Item number M1: “If the study or study protocol was prospectively registered, state the registration platform and provide a link. If the exercise was not registered, this should be stated. Recommended to include the date of registration” [56], p. 11) Item number R2: “Explain any deviations from the study protocol, and why these were necessary. For example, addition of panel members during the exercise, number of consensus steps, stopping criteria; report the step(s) in which this occurred” [56], p. 18)	Can global consensus be arrived at as to the essential components of training for ensuring confident and effective medical practice in remote settings?” [57]	“A protocol for the development of a critical thinking assessment tool for nurses using a Delphi technique” [58]

*Abbreviations ACCORD* ACcurate COnsensus Reporting Document, *BQQRG* Big Q Qualitative Reporting Guidelines, *CONSORT* Consolidated Standards for Reporting Trials, *MMR-RHS* Mixed Methods Reporting in Rehabilitation Health Sciences Research, *PRIOR* Preferred Reporting Items for Overviews of Reviews, *PRISMA* Preferred Reporting Items for Systematic Reviews and Meta-Analyses, *PRISMA-P* Preferred Reporting Items for Systematic Reviews and Meta-Analyses for Protocols, *PRISMA-ScR* PRISMA Extension for Scoping Reviews, *SPIRIT* Standard Protocol Items: Recommendations for Interventional Trials, *SRQR* Standards for Reporting Qualitative Research, *SQUIRE-SIM* Standards for Quality Improvement Reporting Excellence for SIMulation, *STROBE* Strengthening the Reporting of Observational Studies in Epidemiology Statement

^**†**^Examples provided do not all use protocol or reporting guidelines that are included in table. They are examples of protocols that may or may not adhere to these guidelines

## The valuable relationship between a protocol and a reporting guideline

A protocol is a *living* document for the duration of a project [1] – it is not set and forget. Much like a cook-book recipe, it should be frequently referenced throughout a project to ensure methods are being adhered to, and where post-hoc deviations exist, they are documented for the final report [19] (it’s ok to halve the amount of sugar, or add some spice if you think the outcome will be better – it just needs to be justified and clearly reported so that others could reasonably use the same methods as you have and can clearly understand each step and decision that was made along the way). The final report needs to be robust. To support researchers and peer-reviewers to accurately, consistently and comprehensively produce a final report, reporting guidelines have been developed for broad categories and specific types of research.

Acceptance and expectation for the use of reporting guidelines is evident in our, and many other fields – in fact over 650 guidelines are now available to support the reporting of different types of research via the EQUATOR Network (https://www.equator-network.org/reporting-guidelines/). The CONSORT and STROBE guidelines have been adapted specifically for health simulation research [31], the SQUIRE 2.0 reporting guideline for quality improvement projects has been adapted for simulation-based quality improvement projects as SQUIRE-SIM, and most systematic and scoping literature reviews have used at least part, if not all of the PRISMA and PRISMA Extension for Scoping Review reporting guidelines [45, 49].

Contrary to what may seem logical, reporting guidelines should be used from the beginning of a project – informing each section of protocol development and eventual reporting of project findings [59]. We can consider this as *thinking with the end in mind*: if we don’t remember to collect data about participant demographics in the design and data collection phases, we are not going to be able to fulfil reporting requirements when we are writing up our final report. Some of the more frequently used reporting guidelines are included in Table 1, alongside extracts that feature information relevant to research and review protocols.

## Prospectively publishing and/or registering a protocol

So, now that I’ve convinced you to have a project protocol that is *prospectively* available to the public should you now register it, publish it, or both? This depends on several factors. Benefits and considerations of each approach are indicated in Table 2.

**Table 2:** Considerations for choosing between registration or publication of a protocol

	Benefits	Considerations
Protocol registration	• Publicly available for reference when project report is under review and published • Publicly available for use by other researchers seeking to undertake the same or similar research • Has a unique alpha-numeric identifier or URL that can be referenced in subsequent publications	• Peer-review not formally conducted by content or methodology experts; feedback that may enhance project will not be integrated into a final version before data collection commences • Consider asking colleagues or experts in the field to provide informal feedback before commencing study (for example, in a step-back consultation [25]
Protocol publication	• Publicly available for reference when project report is under review and published • May be publicly available for use by other researchers seeking to undertake the same or similar research • Has a unique alpha-numeric identifier or URL that can be referenced in subsequent publications • Peer-reviewed by content and/or methodology experts who can provide feedback prior to recruitment and/or data collection	• Accessibility to published protocol may be restricted if it is not published with an open access agreement or if readers don’t have institutionally provided access • Team will experience a delay in starting the project until feedback from peer-reviewers has been received and any appropriate changes have been integrated into the protocol

Further considerations include *where* to register or publish your protocol. Several journals publish literature review and primary research protocols. Depending on your topic and field of interest, protocol submissions are considered by editors in much the same way as research reports and are subjected to the same or similar peer-review processes. Registration of protocols is determined by the type of research that is being undertaken. For systematic reviews, the most commonly used platform is PROSPERO (https://www.crd.york.ac.uk/prospero/). For educationally-focused reviews, IDESR is available for protocol registration of different forms of evidence synthesis, including systematic and scoping reviews (https://idesr.org/). For almost all other protocols relevant to health simulation and health professions education research, the Open Science Framework is an open-source platform hosted by the Centre for Open Science (https://osf.io/).

## Reviewing protocols and their subsequent reports

Peer-reviewing a project protocol shares many similarities with peer-reviewing a report. However, the reviewers have a greater burden and a greater opportunity. There is a greater burden to determine if the project is sufficiently justified, resourced and planned and greater opportunity to positively shape and influence project and outcome optimisation [60].

Three key considerations should guide protocol peer-reviewers:

1. Does the protocol provide adequate detail for how and if the research question(s) will be addressed?
2. Does the protocol provide adequate detail to demonstrate feasibility?
3. Does the protocol provide enough detail to allow another research team, to undertake the same (or similar) study and arrive at comparable conclusions? [61]

As we move into an era where protocols are routinely made publicly available, the practice of peer-review will shift from reading and critiquing one document (i.e. the project report) to reading and critiquing the project report alongside the registered or published protocol. In many ways, this will make the peer-review process easier and more valuable, as reviewers will be able to determine how the researchers adhered to their protocol, if they deviated from the protocol and if they provide sufficient information and justification for doing so.

## Research as an evolving practice

Health simulation research has matured considerably, shifting from early advocacy-oriented and proof of concept work towards an established field of scholarly and research outputs that articulate a deep appreciation of social dynamics, and context-dependencies. The health professions education field of research has also evolved and advanced over several decades, incorporating new methods, philosophical stances and foci [24]. These related fields have greatly benefited from adopting and experimenting with diverse methodologies and from integrating theory-informed approaches [21, 24]. Sustained calls for improvement in project design and reporting often point to using guidelines to support research processes and reporting [24]. However, there remains a persistent unevenness in the quality of research being undertaken and the quality of the reporting of this research [22–24, 62].

As argued throughout this paper, ensuring projects have publicly available protocols, either through the registration or publication routes, is one way to advance our research practice. But as with all changes in practice and expectations, this will not come without consequences. It is likely that the process of undertaking a research project may feel slower with the addition of a process that is not currently routine. It is also likely that peer-reviewers will more frequently be asked to review protocols, providing expert input to projects at a phase that, again, is not routinely supported by external review. To support these evolutions in research practice, journal editors have an opportunity to provide guidance regarding protocol peer-review to researchers and reviewers, and we all have an opportunity to set the reasonable expectation that research project reports are accompanied by an a priori protocol.

## Conclusion

Scientific enquiry is not a destination – it is a journey with well-trodden roads and avenues through which we can innovate, and ample opportunity for improvement. One such improvement should be how we approach constructing and publicising the foundations of our research projects. Research and review protocols that are publicly accessible through registration and/or publication are vital documents for supporting high quality, valuable research. They are a mechanism for supporting transparency, accountability and rigour; they guard against poor research practice and research waste, and they allow us to expand our learning opportunities through our attention to process, not just results. As a research community, we have scope to do better research, and research for the *right* reasons. This may mean we do less research, but the work that is done will be much richer, and more valuable to our society for the care we take to ensure we are doing it well.

## Data Availability

No datasets were generated or analysed during the current study.
